# An examination of relational dynamics of power in the context of supported (assisted) decision‐making with older people and those with disabilities in an acute healthcare setting

**DOI:** 10.1111/hex.13750

**Published:** 2023-03-14

**Authors:** Deirdre O′Donnell, Carmel Davies, Lauren Christophers, Éidín Ní Shé, Sarah Donnelly, Thilo Kroll

**Affiliations:** ^1^ UCD Centre for Interdisciplinary Research, Education and Innovation in Health Systems (IRIS), School of Nursing, Midwifery and Health Systems University College Dublin Dublin Ireland; ^2^ Graduate School of Healthcare Management Royal College of Surgeons in Ireland Dublin Ireland; ^3^ School of Social Policy, Social Work and Social Justice University College Dublin Dublin Ireland

**Keywords:** acute healthcare, capacity, dementia, disability, health decision‐making, older people, supported decision‐making

## Abstract

**Introduction:**

Supported (assisted) healthcare decision‐making (ADM) focuses attention on how people with disabilities, including cognitive impairments, can be best supported to make decisions about their health and social care on an equitable basis with others. Meaningful implementation of legal frameworks for ADM challenges long‐held presumptions about who has access to valued decision‐making resources, influence and power within a particular socio‐cultural setting. This study aims to explore the relational power dynamics around ADM with older people in acute care settings.

**Methods:**

This study adopts a critical hermeneutic approach to qualitatively explore the lived experience of ADM from the perspectives of Health and Social Care Professionals (*N* = 26). This is supported by an exploration of the experiences of older people (*N* = 4), older people with a diagnosis of dementia (*N* = 4) and family carers (*N* = 5).

**Results:**

We present three themes of data analysis that represent three spaces where the relational aspects of power in ADM are manifested. The first space, *centralising decision‐making power within multidisciplinary teams* identified the privileging of physicians in traditional hierarchical leadership models that may lead to the implicit exclusion of family carers and some Health and Social Care Professionals in the ADM process. *Privileging cognitive and communication competence* identified a tendency to attribute decision‐making autonomy to those with cognitive and communication competency. The final space, *balancing the duty of care and individual autonomy*, recognises acute care settings as typically risk‐averse cultures that limit autonomy for decisions that carry risk, especially for those with cognitive impairment.

**Conclusion:**

Findings indicate the need to address cultural sources of power operating through social norms premised on ageist and ableist ideologies. It is necessary to challenge institutional barriers to meaningful ADM including positional power that is associated with hierarchies of influence and protectionism. Finally, meaningful ADM requires resistance to the disempowerment created by structural, economic and social circumstances which limit choices for decision‐making.

**Patient or Public Contribution:**

A public and patient involvement panel of older people were consulted in the development of the grant application (HRB: APA‐2016‐1878). Representatives from Alzheimer's Society Ireland and Family Carers Ireland were steering committee members guiding design and strategy.

## 1 INTRODUCTION

The United Nations Convention on the Rights of Persons with Disabilities (UN CRPD) provides an international legislative basis for the human rights of all persons, including those with a disability, to participate as fully as possible in all decisions which affect their lives.^1^ Following the UN CRPD, some countries have adjusted their legislation to include legal mechanisms for supported decision‐making (SDM). SDM focuses attention on how people with disabilities, including cognitive impairments, can be best supported to make decisions about their health and social care on an equitable basis with others. In Ireland, the Assisted Decision‐Making (ADM) (Capacity) Act (2015) was prompted by the ratification of UN CRPD and followed a re‐evaluation of pre‐existing guardianship, trusteeship and mental health laws.^2^ It places an onus on Health and Social Care Professionals (HSCPs) and decision supporters to maximise individuals' decision‐making capacity and to ensure that their will and preferences are at the centre of those decisions.^2^, ^3^ The term ADM is used here as a synonym for SDM. It refers to the active decision‐making of people with disabilities in their health and social care and all forms of decision‐making support.

The challenges of translating legal frameworks for ADM with people who have cognitive or communication impairments into health and social care practice are well documented by predecessor states.^4^, ^5^, ^6^, ^7^, ^8^, ^9^, ^10^, ^11^ As Ireland plans for the commencement of this act, it is necessary to obtain a nuanced understanding of complex implementation factors, including culture and leadership, social environment, resourcing and education of HSCPs and the general public.^12^, ^13^

Implementation of ADM legislation is likely to be particularly challenging in the context of acute care when older people, particularly those with complex medical conditions, present with fluctuating or stable, cognitive impairments.^3^, ^8^ Early transition to palliative care may be considered an important element of responsible care planning with some older persons in acute care.^14^, ^15^ However, Voumard et al.^16^ note that the development of cohesive synergy between geriatric medicine and palliative care is challenged by increasing fragmentation, specialisation and sectorisation in healthcare. Furthermore, early transition to palliative care in acute settings continues to be challenging in Ireland, prompting policy recommendations for improved staff training, education and practice guidelines.^17^, ^18^

Older people may also experience vulnerability to abuse, including coercive control and neglect.^19^ Elder abuse is a complex and multifaceted phenomenon that often presents ethical challenges for HSCPs in reconciling the autonomy and self‐determination of older people with issues of capacity, risk and vulnerability.^20^, ^21^ Supporting older people to realise their will and preferences requires highly skilled HSCPs as well as the provision of meaningful choices through access to valuable decision‐making resources. This is crucial to addressing the tension between protectionism and empowerment presented by a human‐rights approach to ADM.^21^, ^22^, ^23^

Meaningful implementation of ADM challenges long‐held presumptions about who has access to valued decision‐making resources and influence within a particular socio‐cultural setting. Smith^24^ describes the hegemony of ‘health people’ as being premised upon legitimising language, knowledge, methods and power to define health or illness. Hegemony and the status quo are supported by ideologies such as ageism or ableism, which are often internalised as accepted social norms and values. Arguably, internalised paternalistic norms associated with duty of care and beneficence remain legitimising forces for inequitable power relations in healthcare.^25^

This study adopts a sociological understanding of power whereby ADM is considered a disruptive force of the status quo through challenging normative assumptions supported by ideologies of ageism or ableism. McCartney and colleagues^26^ present a theoretical framework for the exploration of sources of power in the context of the spaces in which power relationships are manifested. They argue that an examination of socio‐political processes of contested power dynamics is critical to understanding and identifying opportunities for challenging the root causes of health and social inequalities.

This study aims to explore the relational power dynamics around ADM between older people, family carers and HSCPs in an acute healthcare setting.

## 2 METHODS

This exploratory, qualitative study was guided by a critical hermeneutic approach which placed analysis in the broader socio‐cultural context of acute care of older people and explicitly addresses issues of power.^27^, ^28^

### 2.1 Participants and recruitment

The study received ethical review and approval from the University College Dublin Human research ethics committee in 2018 (REC reference LS‐18‐73‐ODonnell). The study participants were HSCPs working in the acute care of older people, older people (including those with a diagnosis of dementia) and family carers (see Tables 1, 2, 3, 4). A convenience sample of HSCPs was drawn from two large urban academic teaching hospitals in Ireland. Project steering committee collaborators, HSCPs from each of the hospitals (three consultant geriatricians, one advanced nurse practitioner and the end‐of‐life care coordinator), acted as links between the research interview team (D. O. D., É. N. S., S. D. and F. F.) and potential participants, facilitating recruitment. The research team attended multidisciplinary care‐of‐the‐older‐person team meetings in the two sites and presented on the study. HSCPs who expressed interest were emailed a participant information sheet and invited to participate.

**Table 1:** Overview of participant characteristics (*N* = 39).

Group	Characteristic	Inclusion criteria	Male	Female
Hospital A *n* = 11	Doctors (*n* = 5)	Working in acute care in care for older person service	1	4
	Nurses (*n* = 0)			
	Allied Health (*n* = 6)		1	5
Hospital B *n* = 15	Doctors (*n* = 5)		0	5
	Nurses (*n* = 4)		1	2
	Allied Health (*n* = 7)		0	7
Older people *n* = 8	Older people (*n* = 4)	An older person with experience of acute care admission in the previous 6 months.	4	0
	People with a diagnosis of dementia (*n* = 4)		2	2
Family carers *n* = 5	Family carers (*n* = 5)	Family carer with experience in accompanying an older person to an acute care setting.	0	5
Total *n* = 39			9	30

**Table 2:** Participant characteristics for family carers (FCs) (*N* = 5).

ID	Age of carer	Years in a caring role	Diagnosis of the recipient	Relationship with the recipient
FC1	67	11	Neuromuscular disorder	Wife
FC2	64	23	Learning disability	Older sister
FC3	38	4	Dementia	Daughter
FC4	62	35	Autism	Older sister
FC5	33	2.5	Cancer	Daughter

**Table 3:** Participant characteristics for people with a diagnosis of dementia.

ID	Age	Diagnosis
OPD1	69	Alzheimer's disease
OPD2	68	Alzheimer's disease
OPD3	55	Lewy body dementia
OPD4	74	Alzheimer's disease

Abbreviation: OPD, older person with dementia.

**Table 4:** Participant characteristics for older people (OP).

ID	Age	Reason for admission
OP1	80	Melanoma
OP2	76	Stroke
OP3	74	Gallstone sludge
OP4	72	Ear infection

Convenience sampling was also used to recruit older people, including those with a diagnosis of dementia and family carers. Recruitment was facilitated via our public and patient representative organisations: Family Carers Ireland, the Alzheimer's Society of Ireland and SAGE Advocacy. Each third‐party organisation promoted the study via different forums (member meetings, emails, newsletters, social media, text messages and word of mouth). Inclusion criteria were older people (self‐identified) who had a recent experience of an acute care admission or family carers who had a recent experience accompanying an older relative/friend to the hospital.

To facilitate informed and voluntary consent to participate in the study, those who indicated via a third party, their willingness to participate in the research were provided with a participant information leaflet and consent form. The production of written communication material followed the National Adult Literacy Guidelines for accessible communication. Participants were encouraged to read these documents on their own time and share them with a nominated decision‐making assistant/supporter, where relevant. They were offered the opportunity to discuss the study with a nominated representative of the third‐party organisations supporting recruitment, a member of the research team and/or their designated decision‐making assistant at any point before or while giving consent to participate.

### 2.2 Data generation

Interviews took place between January and June 2019. Interviews with HSCPs took place in the respective clinical locations, while the older people and family carers were interviewed at home or an alternative place of their choice. The interviewees were asked to narrate a recent acute care experience with follow‐up questions eliciting further narrative reflection. The Alzheimer's Society of Ireland advised that some older people with a diagnosis of dementia may have challenges in narrating a personal experience. With their guidance, we created a scenario as a prompt if required by the interviewees. The scenario involved an older person identified as having anaemia while receiving treatment in a hospital for an infection. The participant was brought through the scenario in stages and prompted to draw upon their own experiences of acute care to reflect upon decision‐making contexts and situations presented in the scenario. Only one interviewee required this innovation. All interviews were audio‐recorded with permission and subsequently transcribed.

### 2.3 Analysis

The current study adopts a critical hermeneutic approach to analyse the socio‐cultural factors that influence meaningful ADM implementation with older people in acute healthcare settings. Critical hermeneutics recognises that all interpretation is dependent upon relational social and historical power dynamics that shape lived experience.^27^, ^28^ In our analysis, we examine contested power dynamics at the intersection of ageism and ableism. To support the appraisal of the confirmability of our findings we reflexively acknowledge our conceptualisation of power as a social and relational dynamic that sustains regimes of inequality, including health inequality, although in the presence of resistance and challenge.^26^ This theoretical understanding of contested power informed our interpretation of the findings. Our analysis focuses on the lived experience of relational power dynamics in the context of ADM with older people in acute care settings. This included dynamic and relational experiences of inclusion/exclusion, silencing/voicing and choice/coercion.

Weekly meetings over a 3‐month period (approximately 10 meetings) were held between researchers undertaking analysis (D. O. D., L. C. and C. D.) allowing for peer‐debriefing to develop an understanding and interpretation of the data. Inductive thematic coding of the data was undertaken via a licensed version of NVivo 12 Pro. We used a two‐step process: (1) initial open in vivo coding and (2) aggregation of in vivo codes and abstraction into higher‐order themes. All the interviews were coded into the lower order in‐vivo codes by one researcher (D. O. D.). These were then verified by a second researcher (L. C.). Discrepancies between the two researchers were discussed and resolved at weekly meetings with a third researcher (C. D.). At these meetings, the second‐order thematic clusters (step 2) were agreed upon through consensus. When all of the initial open in vivo codes were incorporated into the higher‐order themes it was felt that data saturation had been achieved. There were no emerging in vivo codes from the data that were not already accounted for in the thematic clusters.

A group meeting with HSCP members of older person multidisciplinary teams was organised in each of the hospital sites. The interview participants were included in these sessions alongside other nonparticipant HSCPs. At these meetings, the initial study findings were presented for discussion and confirmation. A summary document of the thematic analysis was sent to each of the non‐HSCP interviewees. Follow‐up phone calls or home visits were made to discuss the tentative findings and gather their feedback. Upon completing the respondent confirmation process, a broader research team meeting was held in which participant feedback was discussed and integrated into the inductive thematic structure (D. O. D., C. D., L. C., É. N. S., S. D. and T. K.).

The following identifiers are used to present the findings: FC (family carer), OP (older person), OPD (older person with dementia), MD (medical doctor), Ns (nurse), SW (social worker), SLT (speech and language therapist), OT (occupational therapist), Dt (dietitian), PT (physiotherapist).

## 3 RESULTS

We present three spaces where the relational aspects of power in ADM are manifested in the data analysis:

1. Centralising of power and influence within multidisciplinary teams.
2. Privileging cognitive and communication competence.
3. Balancing duty of care and individual autonomy.

### 3.1 Centralising decision‐making power within multidisciplinary teams

Being valued as a member of an inclusive care team was important to family carers. They saw themselves as an advocate, the person's ‘voice’ (FC3) and the key to enabling will and preference (FC5). In their accounts the older people spoke about wanting their family carer to be involved in care planning conversations: ‘if there was a decision to be made, they could help me discuss the possible outcomes’ (OPD1). They also noted the critical importance of older people being at the centre of decision‐making:

No longer can people talk around the patient you know to a third party whether it's a daughter or a wife. But they should be involved now it doesn't mean to say they fully understand what is happening but at least they are a human being it's their health it's their future. (OP2)

This older person with dementia described the value of being listened to and how that shaped their relationship with their care provider and their sense of self‐esteem:

Right there and then I knew I could trust him I knew I could talk to him he wasn't one of these guys that is I know this I know all […] the very fact that he just took a minute and listened to what I wanted and what I thought […] it gave me such faith in that doctor (OPD3)

The HSCPs frequently acknowledged the importance of inclusive decision‐making which empowered older:

I've seen a few patients being involved in the family meetings. I think it's a nice space for the patient to feel supported and comfortable […] it was a good way of him feeling actually like ‘I can speak to them and tell them that I actually want to go home'. (Ns2)

Despite a general acknowledgement of the importance of including family carers and older people in decision‐making, the participants frequently recounted incidences of exclusion. For example, this family carer spoke about being dismissed by a physician who was treating their mother who had a diagnosis of dementia:

I remember a few times him coming in and knowing that she had dementia and him with a team […] trying to get answers out of her and her not able to give any answers and I remember me asking him questions of what did he mean and he wouldn't speak to me […] He totally dismissed me. (FC3)

A culture of care that centralised decision‐making power with HSCPs had the effect of diminishing the autonomy and dignity of an older person. This was powerfully described by a physician recalling older people being coerced into residential care against their expressed will and preference:

A patient […] whose arm is being twisted basically and who was traditionally told all sorts of rubbish about you know […] will go and try it for maybe a week or two of convalescence and see how it goes, knowing that that was the plan going forward. (MD6)

The participants described a cultural legacy that historically privileged the knowledge of a physician and legitimised their authority in care decisions:

I would say an awful lot of people are afraid of the consultants afraid of the doctors terrified to ask them anything in case oh if I say something […] they will have it in for me you know. That is old fashioned fear is still there with the elderly people. (OPD3)

The privileging of physicians within a hierarchical leadership model was also recognised as creating differential access to decision‐making influence across professions. This was particularly evident in discussions about how a person's capacity is evaluated:

It's still kind of not considered culturally here or appropriate for me to say that the man has capacity, it's still very much viewed as that's the consultant's decision. (OT2)

The narratives of the HSCPs revealed a tension with interprofessional collaboration that existed within some teams and which largely depended upon the leadership style of the particular consultant:

…some consultants would be like that and be the decision‐maker, others would rely hugely on the occupational therapist and the social worker in that kind of scenario. (MD9)

They described their strategies for negotiating and asserting influence within hierarchical organisational structures. For example, a social worker described their strategy of allying themselves with the consultant to assert their influence in the care planning process:

But it's very hard to bring something in when you are being blocked at the top. So you really it's really about getting the doctors involved I think once they are on board then you are grand. (SW4)

The multidisciplinary teams managing the care of older people were generally felt to be further evolved than other specialities in creating more cohesive interprofessional collaboration. A dietician reflected upon how their insight into mood was welcomed by an older person's care team and contrasted this with other specialities:

But I think geriatrics was appreciative of the information if I reported a low mood the team would investigate whereas if I reported a low mood in other specialities they would probably would just tell me to focus on my own work. (Dt2)

This theme has explored positional power dynamics associated with hierarchy and interpersonal relations that influence how ADM is undertaken within acute care settings.

### 3.2 Privileging cognitive and communication competence

The challenge of assessing the capacity of people who require support to weigh information and/or consistently express their preferences was noted by HSCPs.

And if the patient is clear on their views then the situation becomes we try and always go along with the views. If they are inconsistent well then that is a different story you know if one day they want to go home the next day don't want to go home… (MD10)

A lack of understanding of how to support a person's communication and decision‐making competence may result in an association between autonomous decision‐making with cognitive and communication abilities. A dietician recounted the story of a person with advanced dementia who was refusing food:

She clenched her teeth whenever we tried to bring the spoon to her […] now and again her eyes were open she would clench her teeth and the daughter at one stage had tried to shove some ice cream into the lady's mouth and she spat it out. So that was the only form of communication. (Dt2)

In the dietician's account, the person went on to have a nasogastric tube inserted at the request of the family who was reluctant to engage with end‐of‐life care. This story demonstrated the necessity to understand behavioural rather than vocal expressions of autonomy:

I have seen so many people refusing food which is a sign of end of life in dementia. […] we had no obvious signs that like she wasn't pulling her NG tube […] But I think she was communicating with me […] which showed to me that this was inappropriate. (Dt2)

Participants' accounts revealed that there was often confusion amongst professionals regarding the distinction between cognition and capacity, which was particularly complicated for people with a communication disorder. They described the use of assessments of cognition as indicative of decision‐making capacity:

I think they're still holding on to the, well if they have an MMSC of this therefore they are cognitive impaired, can't make decisions and it's never been that black and white. (SLT 2)

A speech and language therapist spoke about working with a person who had aphasia to support their communication competence thereby revealing capacity despite being previously misdiagnosed and ‘written off’ as having dementia:

We were able to take time out to bring her to the department to a quiet environment to kind of diagnostically work with her and look at her strengths and weaknesses from a communication point of view. And then train team members and family members to interact with her because people had I guess in a sense written her off as having or most likely dementia just before we diagnosed. But people thought she was really confused and then when she was getting agitated and upset they thought it was because she was agitated and upset. When it wasn't, it was because she had so much to say and couldn't say it. (SLT3)

Family carers were revealed to be an important element of the structures to support autonomous decision‐making, particularly in ascertaining life‐course values and preferences. For example, a family carer spoke about engaging an HSCP in discussions about the withdrawal of active management of their husband, who was experiencing dementia and a cognitive‐behavioural disorder associated with advanced Parkinson's disease:

It wasn't really a major decision because by that time we had talked it over here at home […] he worked in a law office all his life so he knew, his brain was smart enough on good days, but we had talked about it, you know, how long do you prolong it. He was a Christian man, he knew he was going to heaven. […] It wasn't as if he was afraid to die. (FC1)

The participants as they spoke about assisting decision‐making through effective communication. They described ‘simplify(ing) the language’ (NS3); changing their communication approach: ‘we've always had to kind of adapt the way we ask questions’ (SLT1); using communication aids: ‘providing the teams with books of photos, pictures, imagery, strategies to use, for the rest of the team to use in communication with the patient’ (MD6); paying attention to non‐verbal cues: ‘reading what they look like during therapy’ (PT1) and repeating information to allow for its absorption by the patient: ‘it's slow it's repeated discussion after discussion very much the same content but it's just bringing people to that slow realisation’ (Ns3). They also individualised their approach to people to allow for fluctuating capacity: ‘always saw him at a very good time of day for him’ (MD6). Of utmost importance, however, was their recognition of the necessity for time and a focus on the goals and wishes of older people. This work was recognised in the narratives of older people and family carers as central to building strong therapeutic relationships and facilitating autonomy:

He spent probably two hours with me the first day on my own and he probably spend an hour with (family carer) and myself the following day and he kept asking is there any questions you want to know? (OPD3)

The theme has revealed mechanisms for supporting decision‐making capacity which challenges ableist and ageist assumptions regarding autonomy.

### 3.3 Balancing duty of care and individual autonomy

The HSCP participants spoke in great detail about the challenge of balancing an older person's will and preferences against their insight into the risks associated with a decision. They spoke of ‘really agonising over it’ (MD5), ‘measuring the balance’ between risk and a person's will (SW1) and assessing whether the ‘right decision is being made’ (SW2). Participants noted that the acute setting was characterised by a culture of risk aversity which was underpinned by normative ideas about ‘duty of care’ and ‘medical best interests’. These normative concepts were associated with ideologies of paternalism and protectionism:

…we have all worked on best interest for years. And you know I suppose there is an element of if I am treating a patient I would like to think that I would treat them the same way I would treat my own relatives you know. […] I do have a slight fear that we may be so heavily on will and preference that we almost throw the baby out with the bathwater. (MD9)

Some participants spoke about a shift towards a more risk‐tolerant culture, which would allow people to succeed in positive outcomes for potentially risky decisions. ‘I think that there needs to be kind of a shift in mentality to empowering them. And going supporting them instead of trying to take away their independence’ (SLT3). However, in advocating for an older person's decision‐making capacity, they often found themselves at the centre of conflicting power dynamics played out in a culture of physician accountability for decision‐making and protectionist concepts of duty of care:

So it's like would you prefer to be in front of the medical council, or would you prefer to be in front of a court having to explain why you, you know deprived somebody of their liberties and their constitutional right to be free? (MD8)

The HSCPs spoke about being afraid of making the wrong decision and a fear of litigation which was particularly evident when a person's decision conflicted with what HSCPs considered to be their medical best interests or what family members believe is safe:

If you know that there is a family member who I would say would take litigation like at the first drop of something going wrong […] Really careful yes and then also be quite confident in sort of saying no like and going against her and that was quite you know intimidating. (SW1)

Collaborative multidisciplinary working, which adopts a functional approach to decision‐making, was seen as a mechanism to resist normative protectionist ideas about risk and safety:

I suppose her family had ideas about what was safe for her and they wanted her admitted for convalescence so that they could create a scenario where she was supervised twenty‐four seven. And she did not want this. And even though she had obvious problems with her memory, she did have fleeting capacity […] So together with the occupational therapist we did some functional testing. And together we supported her decision that she wanted to return to her own home, rather than the family's decision. (MD8)

An under‐resourced social and community care context created a pressurised environment for the HSCPs: ‘So there's a huge gap between what people want and what they can have, unfortunately’ (MD6). In its most negative form, this manifested as a person being persuaded into a decision that was not aligned with their preference, but which was practically realisable: ‘We actually don't have anything alternative sometimes to offer them’ (MD10).

In the following narrative, a physician describes working to support an older person's preference to return home in the context of inadequate community and social care provision, which reduced the choices available for the older person and their family. The situation was associated with the ageist and ableist language of ‘burden’, which operated to demoralise the older person by diminishing their right to autonomous decision‐making:

… I don't want to be a burden to my daughter. There's always the sting in the tail isn't there with, ‘I don't want to be a burden to my daughters. (MD7)

Participants spoke about the personal conflict they experienced in reconciling the will and preferences of older people with the needs and well‐being of over‐burdened family carers who were compensating for poor social care provision:

…it's hard not to listen to that person because […] maybe they are completely exhausted they have their own personal life they are working full time. (Ns4)

This social context for decision‐making was experienced differently by older people depending on their access to buffering resources such as private wealth and/or family care which were accumulated over a lifetime. This physician's narrative reveals the impact of buffering resources to empower some people to realise their will and preference:

She was able to afford to pay for essentially twenty‐four‐hour care. Which isn't a position that a lot of people find themselves in but I suppose she had that luxury […] in the end we were able to comply with her wishes […] She could be supported by her nieces one living down the country used to come up every so often […] And is loving living at home and has done very well. (MD10)

This theme has explored the tension between protectionism and patient autonomy which was exacerbated by inadequate social and community care provision.

## 4 DISCUSSION

Shared clinical decision‐making, with the inclusion of decision‐supporters where necessary and which supports the will and preference of older people, has been recognised as a core competence of interprofessional collaboration in the care of older people.^29^ Our participants identified meaningful ADM within inclusive MDTs as shifting the paradigm towards greater collaboration and devolution of influence on decision‐making, particularly concerning functional capacity assessment and the inclusion of family carers in care planning decisions.

Conceptualisations of shared decision‐making have traditionally privileged the idea of individual expressions of autonomy whereby influence is shared between a treating physician and the autonomous patient.^10^, ^30^ Focusing on individual expressions of autonomy privileges those who have the cognitive and communication competency to represent their will and preferences in decision‐making. This is evidenced in processes of advance care planning (ACP) whereby individuals are supported to establish decisions about future care that may take effect following the loss of decisional capacity. The evidence for the benefit of ACP remains mixed and is particularly challenging in the context of acute care where the conditions for ACP are rarely reflected in practice situations.^31^, ^32^ This was highlighted in the narratives of our participants which focussed on the microdecisions of everyday acute care which involve aspects of discharge and care planning rather than end‐of‐life treatment decisions.

A privileging of cognition and articulacy in the context of engaged research has previously been described as an act of bureaucratic violence created by institutional structures and systems associated with the habitus of research culture.^33^ Our study findings illustrate that decision‐making in acute settings is undertaken within its own distinct habitus which creates implicit and explicit exclusion of those who are less able to articulate an autonomous choice through the use of language. Our participants' narratives highlighted the necessity to challenge the privileging of articulacy which is central to the cultural habitus of acute care through a recognition of the interdependent and relational aspects of decision‐making.

ADM invokes a paradigm shift in its view of autonomy as being expressed interdependently. Building on pedagogic theories of cognitive development,^34^ we depict ADM as necessitating a process of scaffolding capacity through interdependent expressions of values and preferences. This challenges an ageist and ableist status quo as the presumption of capacity places an onus on systems of care to provide this scaffolding thereby enabling contexts and resources necessary to support insight and decision‐making. Interprofessional collaboration, supportive communication techniques, functional capacity assessment and the inclusion of decision supporters in care planning are features of this scaffolding.

Meaningful engagement with ADM encouraged our participants to interrogate what a duty of care entailed and how their understanding of ‘best interests’ could be reconciled with a person's will and preference. In this way, their deliberations forced into view implicit and internalised norms that may have been hidden by legitimising language and authority associated with risk aversion, accountability and protectionism.^24^, ^25^ These internalised norms are evident when ‘medical best interests’ are conceived as somehow distinguishable or separate from a patient's will, preferences or values.

There was a strong sense from the narratives of the HSCPs, particularly from the physicians that they were unsupported in making highly complex judgements when balancing risk with autonomy. This was associated with a culture of protectionism which was sustained by inadequate social resourcing, which often provides the context for acute care decision‐making.^8^ This context influences how HSCPs can measure the balance between risk and a patient's will and preference by limiting the choices older people can have to realise their autonomy.

Participant narratives depicted a hegemonic distribution of power or influence overvalued social resources, which privileges those with the structural advantages associated with life course accumulation of wealth, education, or social capital. ADM represents a disruptive challenge because it exposes the hegemony of an ageist and ableist state responsible for inadequate social resourcing, which would tackle the agents of social circumstances that engender dependency amongst those with complex needs.

### 4.1 Implications for policy and practice

The results have implications for health systems implementing legislative frameworks in line with UN CRPD. This paper draws attention to HSCPs' commitment to ADM in their care practice. However, it is necessary to challenge system‐level barriers to meaningful ADM including positional power‐associated hierarchies of influence and protectionism. Meaningful ADM requires resistance to the disempowerment created by structural, economic and social circumstances which limit choices for decision‐making.

### 4.2 Study limitations

More HSCPs took part in the study than older people or family carers. This may have unfairly skewed the perspective towards narratives from HSCPs. Further, prospective studies may consider appropriate objective measures, case note reviews or ethnographic observations of ADM in practice over time. Furthermore, the inclusion of other healthcare sites and respondent groups would have increased diversity and representation.

## 5 CONCLUSION

ADM/SDM amongst older people and those with a disability is an ongoing progressive effort evolving as legislation translates into practice. Meaningful engagement with ADM challenges ableist assumptions and values. The complex work of scaffolding capacity is a powerful act of resistance to ageist and ableist disempowerment. Healthcare institutions need to garner the spirit of ADM adopted by individual/team efforts and support them with more significant undertakings to eradicate system‐level barriers thereby fostering ADM in all aspects of acute care practice. Otherwise, the existing resistance and struggles experienced by those trying to practice ADM within a system of limitations will lead to further implementation challenges.

## AUTHOR CONTRIBUTIONS

All the authors have made significant intellectual and practical contributions to the overall project from which this paper is derived. Deirdre O′Donnell wrote the original manuscript with the assistance of Carmel Davies and Lauren Christophers. All authors reviewed a draft and inputted feedback or edits. Deirdre O′Donnell, Éidín Ní Shé and Sarah Donnelly undertook the data collection (interviews) with the participants. Deirdre O′Donnell undertook the primary inductive analysis which was validated by Lauren Christophers and Carmel Davies. All authors discussed, refined and validated the analysis. Thilo Kroll is the principal investigator for the project and the grant holder. All authors have read and agreed to the published version of the manuscript.

## CONFLICT OF INTEREST STATEMENT

The authors declare no conflict of interest.

## ETHICS STATEMENT

The study was approved by the University College Dublin Human research ethics committee in 2018 (REC reference LS‐18‐73‐ODonnell). The management of research data has been in line with requirements set out under the European Union's general data protection regulation and Irish data protection legislation. The production of written communication material followed the National Adult Literacy Guidelines for accessible communication.

## Data Availability

The anonymised data that support the findings of this study are available from the corresponding author, upon reasonable request.
